# Supplementary figures and images for: Transcriptional Analysis and Identification of a Peptidoglycan Hydrolase (PGH) and a Ribosomal Protein with Antimicrobial Activity Produced by Lactiplantibacillus paraplantarum
Source: Int J Mol Sci. 2024 Nov 25;25(23):12650. doi: 10.3390/ijms252312650 (PMC11641805; doi:10.3390/ijms252312650)

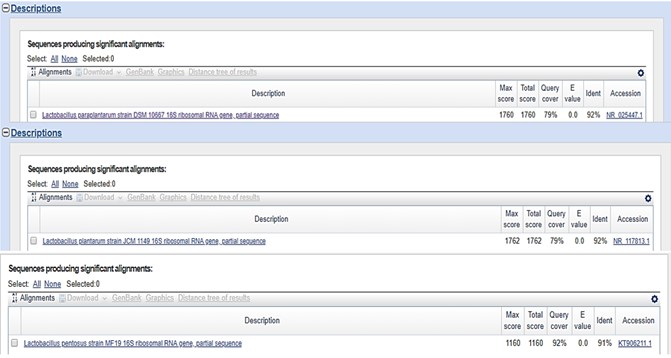

Supplement: Supplementary file 1 [file ijms-25-12650-s001.zip › Figure S1. BLAST results of the 16S rRNA sequence from the strain isolated from salami, analyzed using the NCBI database.jpg]

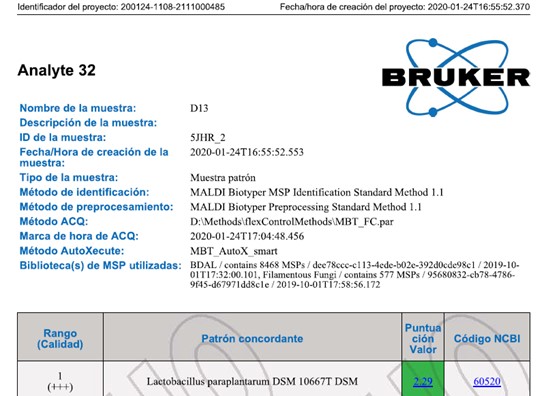

Supplement: Supplementary file 1 [file ijms-25-12650-s001.zip › Figure S2. Identification of Lactiplantibacillus paraplantarum using MALDI-TOFMS and MALDI Biotyper with Score Value Analysis.jpg]

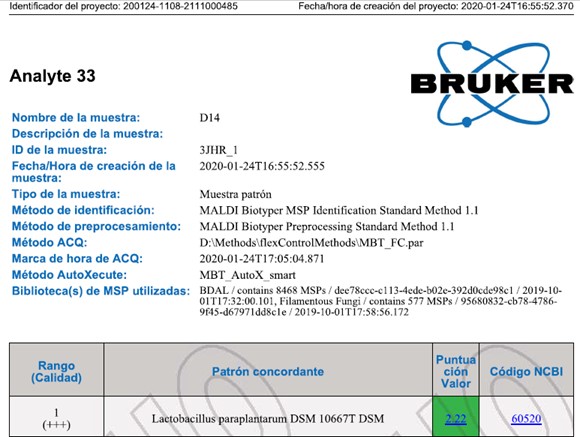

Supplement: Supplementary file 1 [file ijms-25-12650-s001.zip › Figure S2b.jpg]

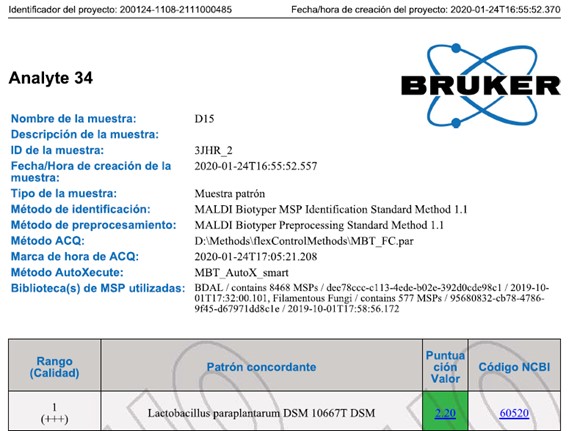

Supplement: Supplementary file 1 [file ijms-25-12650-s001.zip › Figure S2c.jpg]

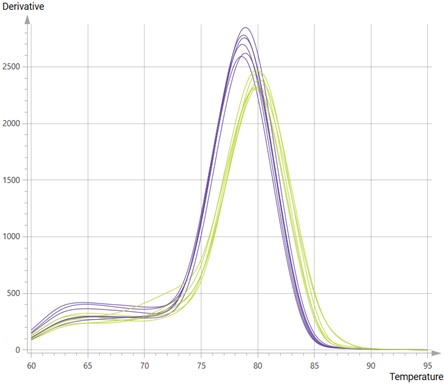

Supplement: Supplementary file 1 [file ijms-25-12650-s001.zip › Figure S3. Dissociation curves of Lacp. paraplantarum cDNA for the condition with the lowest carbon concentration in the culture medium (C1) at 18 h for the RP uL14 gene (green) and.jpg]
